# Anatomical pathways for auditory memory II: information from rostral superior temporal gyrus to dorsolateral temporal pole and medial temporal cortex

**DOI:** 10.3389/fnins.2015.00158

**Published:** 2015-05-18

**Authors:** M. Muñoz-López, R. Insausti, A. Mohedano-Moriano, M. Mishkin, R. C. Saunders

**Affiliations:** ^1^Laboratory of Neuropsychology, National Institute of Mental Health, National Institutes of HealthBethesda, MD, USA; ^2^Human Neuroanatomy Laboratory and Regional Centre for Biomedical Research (CRIB), School of Medicine, University of Castilla-La ManchaAlbacete, Spain

**Keywords:** auditory, memory, superior temporal gyrus, primate, temporal pole, medial temporal cortex

## Abstract

Auditory recognition memory in non-human primates differs from recognition memory in other sensory systems. Monkeys learn the rule for visual and tactile delayed matching-to-sample within a few sessions, and then show one-trial recognition memory lasting 10–20 min. In contrast, monkeys require hundreds of sessions to master the rule for auditory recognition, and then show retention lasting no longer than 30–40 s. Moreover, unlike the severe effects of rhinal lesions on visual memory, such lesions have no effect on the monkeys' auditory memory performance. The anatomical pathways for auditory memory may differ from those in vision. Long-term visual recognition memory requires anatomical connections from the visual association area TE with areas 35 and 36 of the perirhinal cortex (PRC). We examined whether there is a similar anatomical route for auditory processing, or that poor auditory recognition memory may reflect the lack of such a pathway. Our hypothesis is that an auditory pathway for recognition memory originates in the higher order processing areas of the rostral superior temporal gyrus (rSTG), and then connects via the dorsolateral temporal pole to access the rhinal cortex of the medial temporal lobe. To test this, we placed retrograde (3% FB and 2% DY) and anterograde (10% BDA 10,000 mW) tracer injections in rSTG and the dorsolateral area 38*_DL_* of the temporal pole. Results showed that area 38_DL_ receives dense projections from auditory association areas Ts1, TAa, TPO of the rSTG, from the rostral parabelt and, to a lesser extent, from areas Ts2-3 and PGa. In turn, area 38_DL_ projects densely to area 35 of PRC, entorhinal cortex (EC), and to areas TH/TF of the posterior parahippocampal cortex. Significantly, this projection avoids most of area 36r/c of PRC. This anatomical arrangement may contribute to our understanding of the poor auditory memory of rhesus monkeys.

## Introduction

Primates have a surprisingly poor ability to store auditory sensory information into long-term memory (Fritz et al., 2005; Scott et al., 2012). This contrasts with their remarkable capability to form long-term visual and tactile memories (Murray and Mishkin, 1984; Goulet and Murray, 2001). As tested with the delayed non-matching to sample (DNMS) task, visual recognition memory is learned quickly and displays a high level performance at long delays or with many items to remember. In contrast, an auditory version of the DMS/DMNS tasks is very difficult to learn taking many thousands of trials and months to acquire the basic rule and, once learned, performance is poor with monkeys unable to remember more than a single stimulus and only for a few seconds. Similarly, recent behavioral data in humans shows a better ability to remember visual and tactile information than that presented in the auditory modality (Bigelow and Poremba, 2014). As noted in our earlier paper of this series (Muñoz-López et al., 2010), comparison of the visual, tactile, and auditory anatomical pathways might provide us with an explanation as to the difference in recognition memory ability.

The visual system is organized into ventral and dorsal processing streams, with the ventral stream important for object identity and recognition memory (Mishkin and Ungerleider, 1982; Kravitz et al., 2013). The ventral stream is described as organized anatomically in a hierarchical series of connections characterized functionally by the processing of increased stimulus complexity (i.e., 3D objects) at progressively more rostral areas (Mishkin and Ungerleider, 1982; Desimone, 1996; Nakamura and Kubota, 1996; Tanaka, 1996; Kravitz et al., 2013). This processing stream originates in the striate cortex (V1) and courses through the occipitotemporal cortex (V4, TEO) to its anterior temporal target (area TE, Kravitz et al., 2013). Area TE then projects into the memory related areas of the medial temporal cortex, i.e., perirhinal (PRC), posterior parahippocampal (PHC) cortices, and from these to the entorhinal (EC) cortex (Suzuki and Amaral, 1994a,b). Furthermore, tactile information reaches area 35 of the PRC from higher processing somatosensory insular area SII (Friedman et al., 1986). Damage to these rhinal cortical areas results in a severe visual (Meunier et al., 1993; Malkova et al., 2001) but also tactile recognition memory impairment (Goulet and Murray, 2001).

**Figure 1:**
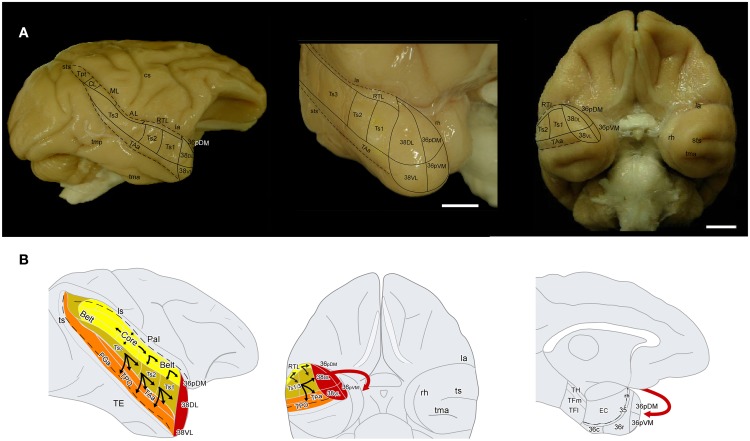
**(A)** Lateral and frontal views of a rhesus monkey brain show the approximate location of the superior temporal gyrus architectonic divisions, including those on the temporal pole. The supratemporal plane can only be appreciated partially because it is located below the frontal operculum within the lateral sulcus. Sulci are indicated with dotted lines. Scale bar: 1 cm. **(B)** Schematic diagram of the auditory ventral stream and the medial temporal cortex. The sulci are partially open to show the divisions of the core and belt areas along the superior temporal plane and of the cortex in the dorsal bank of the superior temporal sulcus. The main aim of this study was to determine the entry of auditory projections to the medial temporal cortex (red arrow).

Ventral and dorsal processing streams have also been described anatomically with respect to audition (Romanski et al., 1999). However, the details on the anatomy and function of the auditory ventral stream are still poorly understood. The auditory ventral stream, thought to be important for processing information about stimulus identity, originates in primary core areas A1/R/RT and courses rostrally in a multistep fashion within the STP and in parallel through the belt areas RM, AL, RTL, RTM (Kaas and Hackett, 2000, see Figure 1). From these rostral belt areas connections course downstream within the parabelt (Ts3), to areas Ts2 and Ts1 on the dorsolateral surface of rSTG (Galaburda and Pandya, 1983; Pandya and Yeterian, 1984) and make their way as far rostral as the dorsolateral temporal pole. Functional imaging studies suggest that the rostral STP and dorsolateral temporal pole are important for processing of complex stimuli such as species-specific calls (Gil-da-Costa et al., 2004; Poremba et al., 2004; Petkov et al., 2008). More specifically, neural responses from the belt/core areas have short latencies to basic acoustic properties of sounds (i.e., frequencies, Tian et al., 2001) while responses in the anterior belt and parabelt have longer latencies, and respond more selectively to complex sounds such as monkey calls (Kikuchi et al., 2010; Perrodin et al., 2011; Fukushima et al., 2014). Taken together, the data suggests a rostrally directed stimulus identity processing stream in STG.

It would appear that direct connections between the auditory association areas of the superior temporal gyrus (STG) with the medial temporal cortex might also underlie recognition memory for sounds. However, monkeys do not appear to have very good auditory recognition memory, at least as tested using conventional tests. This poor auditory memory may be reflected in a difference in the anatomical organization of the auditory system with the medial temporal cortex.

The aim of the present report is to examine the auditory projections from the rostral auditory association areas into areas 35 and 36 of PRC, EC, and areas TH and TF of PHC (see Figure 1B). To investigate this anatomical pathway, we examined first the auditory cortical afferent connections to the dorsolateral temporal pole (area 38*_DL_*) by means of retrograde injections in 38*_DL_* and anterograde tracer injections in the rostral STP and rSTG. The second step was to determine the pattern of efferent projections from rSTG areas and 38*_DL_* to EC, PRC, and PHC by means of anterograde tracer injections into 38*_DL_* and Ts2, Ts3, and RTL.

## Materials and methods

### Subjects

Rhesus monkeys (*Macaca mulatta, N* = 14) of both sexes weighing between 6.0 and 10.0 kg and Cynomolgous monkeys (*Macaca fascicularis, N* = 2, males) weighing between 3.0 and 5.0 kg were used in this study. Five Rhesus monkeys (M3, M6, M7, M8, M12) had forebrain commissurotomy previous to tracer injections and were used as part of a previous study (Muñoz et al., 2009). Experiments were carried out in strict adherence to the Guide for the Care and Use of Laboratory Animals (Clark et al., 1997) and under an approved NIMH Animal Study Proposal and the European Union rules for care and use of animals (UE 86/609/CEE) and the supervision and approval of the Ethical Committee of Animal Research of the University of Castilla-La Mancha (UCLM), Spain.

### Tracers

Surgical details are described previously (Muñoz et al., 2009). Discrete 1 μl injections, of the fluorescent retrograde tracers Fast Blue and Diamidino Yellow (FB and DY, Sigma Chemical CO, St. Louis, MO) suspended in distilled water at concentrations of 3% (FB) and 2% (DY), and the anterograde tracer biotin dextran amine (BDA 10,000 mW, Molecular Probes, Eugene OR) at a concentration of 10% in 0.01 M phosphate buffer, were injected with a Hamilton syringe at a depth of 1.5–2 mm below the cortical surface. A total of nineteen tracer injections aimed at the rostral regions of the STG were analyzed.

### Tissue processing

After a survival period of 2 weeks, the animals were deeply anesthetized and transcardially perfused with 4% paraformaldehyde. The brains were cryoprotected, quickly frozen in −80°C isopentane, and then cut in the coronal plane at 50 μm with a sliding microtome coupled to a freezing stage (Muñoz et al., 2009). Eight, one-in-10 series were processed as follows: two series were immediately mounted onto subbed slides and air-dried, with one series then stored at −80°C, and the second series stored at −20°C in air-tight boxes. Both series were coverslipped with 0.4 M potassium bicarbonate, protected from light, and used for fluorescent retrograde label analysis. A third series was stained with thionin and used for cytoarchitectonic evaluation. A fourth series was processed to visualize BDA transport as follows: (a) 30 min incubation in 1% hydrogen peroxide; (b) incubation in 0.5 μg/ml streptavidin-horseradish peroxidase conjugate (Molecular Probes, Inc., Or.) in 0.05 M Tris buffer (pH 7.6) for 4 h at room temperature and then overnight at 4°C. The two *Macaca fascicularis* brains were processed for BDA visualization following the same avidin-biotin principle, but with the abidin biotin complex (ABC, Vector Ltd., Peterborough, UK) for 2 h at room temperature-; (c) development in 0.025% 3,3-diaminobenzidine tetrahydrochloride reaction (DAB, Sigma Co. St. Louis, MO) with 0.075% hydrogen peroxide and 0.1–0.2% nickel sulfate in 0.05 M Tris buffer (pH 8.0) to intensify the staining and minimize background (3–5 min). Sections were then mounted, dried, and coverslipped. In two animals, a fifth series was stained with a modified Gallyas procedure to visualize fibers (Gallyas, 1979). The sixth, seventh, and eight series were processed to visualize parvalbumin, cytochrome-oxidase, and acetylcholinesterase used to delimit core and belt auditory cortex boundaries. For parvalbumin immunohistochemistry, primary antibody (1:8000) mouse monoclonal IgG1 lyophilized (Swant, Bellinzona, Switzerland) and secondary antibody (1:200) biotinylated horse-anti-mouse IgG (Vector, Burlingame, CA) were used. Visualization started with streptavidin-horseradish peroxidase conjugate in 0.05 M TBS (pH 7.6) and finalized in 0.025% DAB with 0.075% hydrogen peroxide in 0.05 M TBS (pH 8.0). Cytochrome-oxidase staining was based on the Wong-Riley (Wong-Riley, 1979). Briefly, sections were incubated for 1–2 h at 37°C in 0.4% cytochrome C (Sigma Chemical, Co. St. Louis, MO), 0.1 M PB 7.4 pH with 0.6% DAB, and 0.04% glycerol. Sections were rinsed in 0.1 PB, air dried, and coverslipped. Acetylcholinesterase staining was based on the procedure of Tago et al. (1986) as described in Turchi et al. (2005).

### Data analysis

**Figure 2:**
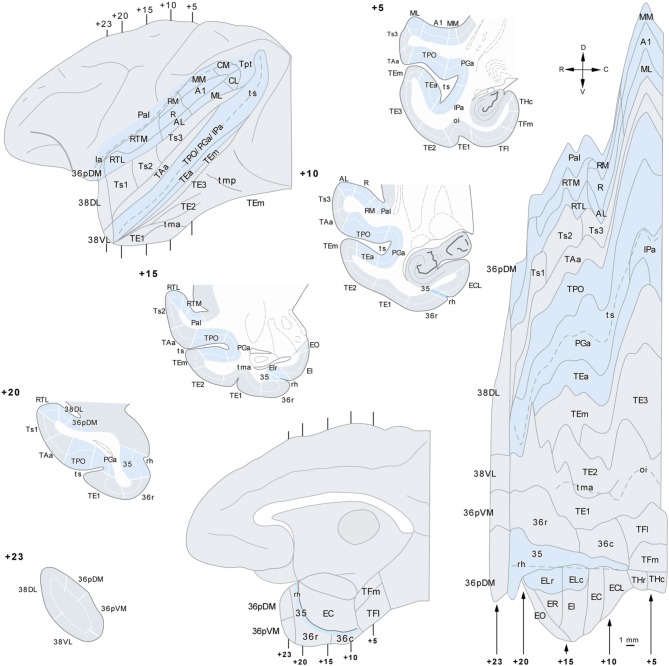
**Lateral view of a standard rhesus monkey brain (left hand side) with the architectonic divisions of the temporal cortex used in this study**. The frontal operculum was removed to show the divisions of the auditory core and belt areas. The gyral surface is represented in gray and fundus and banks of sulci are shown in blue. Coronal sections are arranged from rostral (+23) to caudal (+5) with numbers referring to the distance in mm from the interaural plane. The flat map was used to plot the anterograde and retrograde label.

Individual retrogradely labeled fluorescent cells and anterograde labeled axons in the cerebral cortex in the hemisphere ipsilateral to the injections were plotted from coronal sections 1 mm apart at a magnification of 20× with the aid of an Axiophot Zeiss microscope equipped with a digital video camera (CCD, Optronics, Goleta, CA) and an image analysis system (Bioquant Nova, R&M Biometrics Inc., Nashville, TN). Cytoarchitectonic divisions were analyzed in adjacent thionin sections and were superimposed on sections with anterograde and retrograde label with the aid of a camera lucida. The two *Macaca fascicularis* cases (102BDA and 302BDA) were analyzed with an Olympus B50 microscope and labeled fibers were drawn with camera lucida plotted at a magnification of 20×. Two-dimensional, unfolded maps were constructed for each monkey's temporal lobe following the procedure of Van Essen and Maunsell (1980) (see Figure 2). We used the rhinal sulcus as reference to extend the temporal cortex outline along layer IV/V boundary (Muñoz and Insausti, 2005). Label in the unfolded maps is depicted for layers II–III in green while label in layers V–VI is in black.

## Nomenclature

The approximate location of the architectonic subdivisions of the superior temporal plane (STP), STG, temporal pole cortex (TPC), inferior temporal gyrus (ITG), and the medial temporal cortex are indicated in Figures 1, 2. Rhesus and Cynomolgous monkeys share the cytoarchitectonic features of the areas studied here, with no major differences other than the exact boundary location, and therefore, we used the same nomenclature for both species.

### Temporal pole

The TPC extends anteriorly from the rostral tip of superior temporal sulcus (ts) to the tip of the temporal lobe. The caudal limit medially is near the limen insulae, where it borders with the agranular insular cortex. TPC has been identified as a separate cytoarchitectonic area in humans (area 38 of Brodmann, 1909) and in monkeys (area TG of Von Economo, 1927) and later by Von Bonin and Bailey (1947) (for historical comparative review see Insausti, 2013). Studies of the anatomy of the temporal pole have distinguished an isocortical lateral portion and a medial portion with a more limbic appearance. TPC has been divided into approximately four quadrants according to their laminar organization, with special reference to the presence or absence of layer IV (Moran et al., 1987; Gower, 1989; Kondo et al., 2003). The lateral and medial subdivisions have also been identified in humans, where the lateral temporopolar cortex (TPCl) is related architectonically with the STG, and the medial temporopolar division (TPCm) is closer anatomically to the limbic cortex (Blaizot et al., 2010). In the monkey, our previous cytoarchitectonic descriptions, and that of others, of the medial temporal cortex have included the temporal pole as part of area 36 of PRC given that it shares some architectonic features and has connections with EC (Insausti et al., 1987; Suzuki and Amaral, 1994b, 2003; Blaizot et al., 2004; Lavenex et al., 2004). We have retained the term 36p for the medial side of the temporal pole. Area 36p can be subdivided into a dorsomedial (36_pDM_), and ventromedial portion (36_pVM_). The lateral aspect of the temporal pole resembles the cytoarchitectonics of the adjacent neocortical areas of the STG and ITG, and therefore, we used term area 38 of Brodmann. We further divided 38 into dorsolateral (38_DL_) and ventrolateral divisions (38_VL_).

### Areas 38_DL_ and 38_VL_

**Figure 3:**
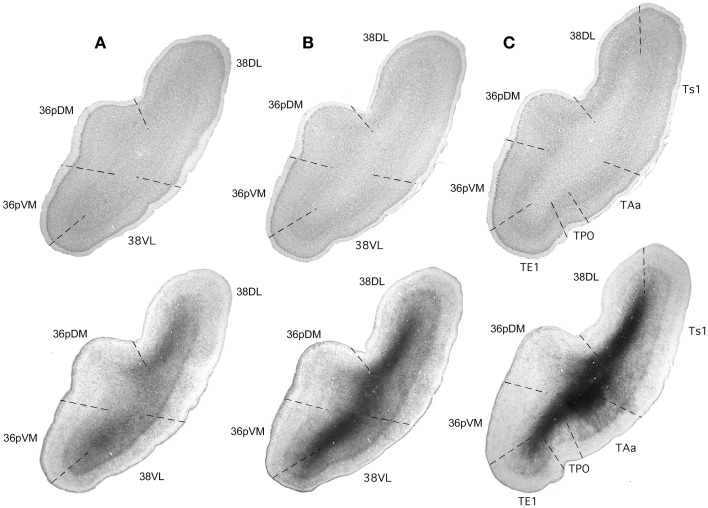
**Rostrocaudal coronal sections (A–C) illustrate the main cyto- and myeloarchitectonic features of the temporal pole cortex**. See text for details.

These two areas lie on the gyral surface of the temporal pole (see Figures 1–3). Briefly, as delineated using thionin stained coronal sections, area 38_DL_ has a characteristic thin layer II rich in small darkly stained cells well differentiated from layer III, a clearly demarcated layer IV, particularly at caudal levels, and fused layers V and VI (Figure 3). In contrast, area 38_VL_ lacks the characteristic darkly stained cells of layer II, but its border with layer III is easily distinguishable. Layer III in area 38_VL_ has the highest cell density and, although modest, the most radial appearance of the adjacent fields of the temporal pole, that becomes progressively more radial at caudal levels (Figure 3). Layer IV in 38_VL_ is more clearly demarcated than in 38_DL_, but layers V and VI still appear fused, as in 38_DL_. Area 38_VL_ is the area within the temporal pole with the closest resemblance to the ventral part of area 36, and further caudally with the six-layered neocortex area TE. Myelin staining confirmed these subareas of the lateral temporal pole. Briefly, as Figure 3 illustrates, the outer portion of layer I in area 38_DL_ contains a band of myelinated axons horizontally oriented. This band becomes narrower in area 38_VL_. Layer IV, typically formed by a dense plexus of myelinated axons oriented perpendicular to the pial surface is very prominent in areas 38_VL_ and 38_DL_ (outer stripe of Baillarger), especially in their caudal portions. This plexus diminishes more medially in areas 36_pVM_ and 36_pDM_ (Figure 3). Caudally 38_DL_borders with parabelt area Ts1 of the rostral STG. Compared with area 38_DL_, area Ts1 has a clearer demarcation between layers II and III, a higher cell density in layer III, a more prominent layer IV, and a less prominent layer V.

### Areas 36_pVM_ and 36_pDM_

These areas lie on the medial aspect of the temporal pole. Area 36_pDM_ is located dorsomedially while area 36_pVM_ occupies the ventromedial portion (Figures 1–3). As with other areas of PRC (36*_r/c_*), area 36_pDM_ is characterized by the clusters of darkly stained cells that appear throughout layer II, although these tend to be less dense than in other more caudal areas of area 36*_r/c_*. There is a wide layer III with a blurred demarcation with layer IV. There is no clear distinction between layers V and VI. In contrast, area 36_pVM_ has smaller cell clusters in layer II, a thinner layer III, and although layer IV is still faint, the layer III and V boundaries are more distinct than in 36_pDM_. The analysis of the myelin staining sections showed that the fiber bundles typical of layer I are wider in both subfields of area 36_p_ relative to areas 38_VL_ and 38_DL_. Area 36_pDM_ has a characteristic band of axons that take an arch-like shape (with one end in 36_pDM_ the other in area 38_DL_, see myelin stained sections in Figure 3). In both, thionin and myelin stained series, layer IV loses its prominence from 38_DL_ toward area 36_pDM_, which lacks layer IV. Area 36_pVM_ contains an incipient layer IV that becomes more prominent near the border with area 38_VL_. Layers V and VI appear fused and darkly stained in the entire TPC, with the exception of area 36_pDM_, which contains a lighter density of myelin stained axons.

### Superior temporal gyrus (STG)

We used the cytoarchitectonic delimitation of areas Ts1, Ts2, Ts3, TPO, PGa, IPa and TAa as defined by Pandya and colleagues (Pandya and Sanides, 1973; Seltzer and Pandya, 1978, 1989, see correspondence with *Macaca fascicularis* in Figure 1 in Muñoz-López et al., 2010). In area Ts1, like in area 38_DL_, layers V–VI appear fused, but in contrast, Ts1 has a very distinguishable granular layer IV. In myelin sections, Ts1 appears more myelinated and exhibits clear a outer stripe of Baillarger compared with area 38_DL_. Caudally, areas Ts2 and Ts3 show better laminar organization and an increase in myelination; inner band of Baillarger begins to emerge. Layers V–VI show a better differentiation in Ts2 than in area Ts1. In area Ts3, the prominent pyramidal cells in layer V make this layer prevail over layer III pyramids and give this area limbic appearance. Medial to areas Ts2/3, area TAa lies entirely in the dorsal bank of the ts. Area TAa has prominent pyramids in layers III (IIIc) and V (Va) and a discrete demarcation between layers V and VI. Area TAa can be distinguished from Ts2/3 by its relatively equal proportion of supra- and infragranular cell layers, and by a characteristic radial arrangement of cells. In myelin sections, the outer bands of Baillarger are darker in Ts2/3 than those in TAa. Area TPO occupies the dorsal bank of the ts. Medial to area TAa, area TPO layer III is broad with many distinct IIIc pyramids, layer IV appears as well-developed although non-columnar, layer V is not as quite prominent as the Va in area TAa, and layers V–VI show a broader space between them than in TAa, owing to the smaller number of sixth layer cells. Area PGa is the third zone in the dorsal bank of the ts, medial to TPO. Rostrally, this area is difficult to locate because of its location in the fundus of the sulcus, but caudally it expands and occupies almost the entire extent of the ts. It is thin cortex with most of its layers only modestly developed. Layer II is thick and layer VI exhibits a characteristic cluster-like arrangement. Area PGa is better myelinated than the adjacent area PG, it has both bands of Baillarger (faint inner one) and a dense plexus of vertical fibers. Inner layer of Baillarger is scarcely visible, but the vertically oriented myelinated fibers are better developed than in area TAa.

Earlier auditory processing areas within the rostral portion of the STP, including core and belt areas (i.e., A1/R, RM, AL, RTL, RTM, ML, MM, CL, and CM) were identified according to Kaas and Hackett (2000). Briefly, core areas (A1/R) lie in the center of the STP and are characterized by its high density of cytochrome-oxidase, acetylcholinesterase, parvalbumin, and myelinated fibers and a prominent layer IV as seen in Nissl stain. There is a progressive decrease of positive staining and of layer IV laterally and rostrally in the surrounding belt areas (AL, RM, RTM, RTL, ML, MM, CL, and CM), and even more so in the adjacent parabelt areas. For the parabelt areas located laterally to belt areas we have used Pandya's architectonic divisions Ts3-1 (Figure 2).

### Inferior temporal gyrus (ITG)

We adopted the architectonic divisions of Von Bonin and Bailey (1947) with modifications (Seltzer and Pandya, 1989, Figure 3). Briefly, within ITG, there are different architectonic areas from medial to lateral: TE1, TE2, and TE3, and two in the ventral bank of the ts; TEm and TEa. There is a progression in the architectonic organization from medial to lateral whereby supragranular layers become more prominent, pyramidal cells in layer IIIc make this layer progressively more distinct, layer IV is gradually more differentiated, and layer VI becomes clearly apparent and differentiated from layer V.

### Medial temporal cortex

In this study, we adopted the terminology of Amaral et al. (1987) for EC architectonics and the terminology of Suzuki and Amaral (1994a,b, 2003) for PRC and PHC with two slight modifications. First, we unified 36rm-36rl under the term 36_r_ and 36cm-36cl as 36_c_, and second, we found an increasingly prominent layer IV caudally in area TH, and therefore we used the term THc to differentiate this region from the more rostral portion, namely THr, in which layer IV is absent.

## Results

### Injection sites

**Figure 4:**
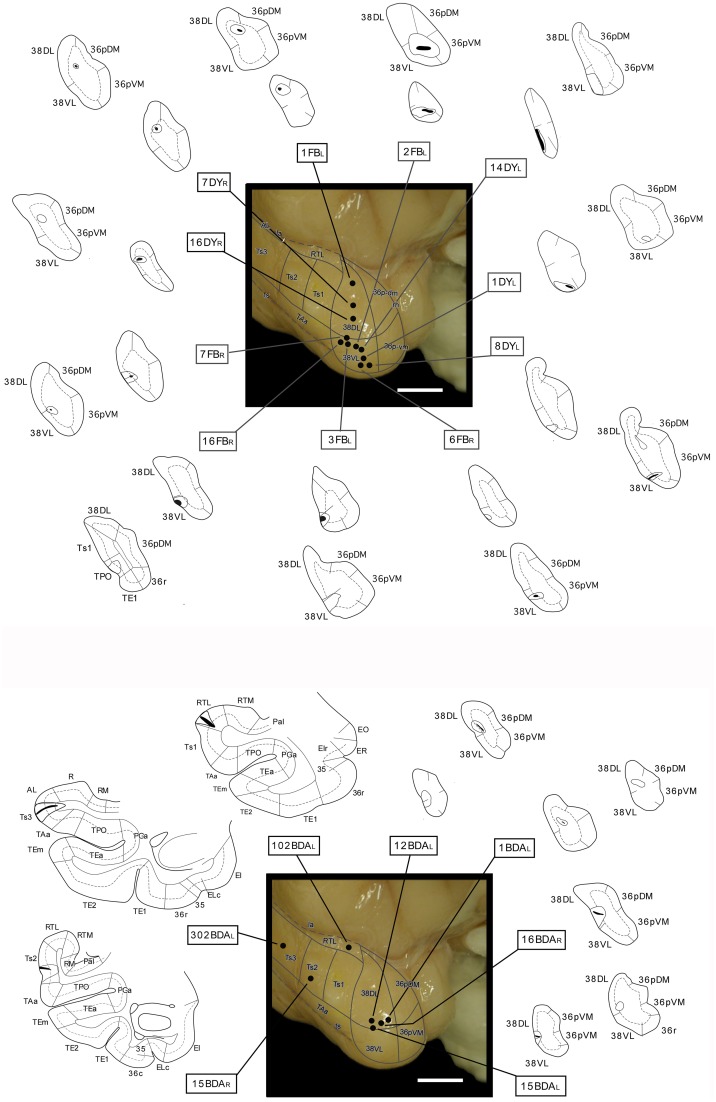
**Location of retrograde tracer injections (FB and DY) in different dorsoventral parts of the lateral temporal pole and location of anterograde (BDA) tracer injections in areas RTL, Ts3, and Ts2, and in area 38_DL_**. The pial surface (solid line) and layer IV (dotted line) indicate the laminar involvement of the tracer injection sites. Tracer injections were placed in both right or left hemispheres (indicated with L and R), but all injection sites are shown in the left hemisphere.

Figure 4 illustrates the location of the 12 retrograde tracer injections at different dorsoventral levels within area 38 that were used to investigate the auditory projections to the temporal polar cortex. Second, anterograde tracer injections in areas RTL, Ts2, and Ts3 were used to examine in more detail the STG projections to the temporal polar cortex and to explore possible direct connections to the medial temporal cortex. Figure 4 also shows the anterograde tracer injections in area 38_DL_ destined to determine the full extent of the projection from this area to the medial temporal cortex.

### Afferent projections from STP and STG to the temporal pole

#### Retrograde injections in dorsolateral lateral temporal pole area 38_DL_

**Figure 5:**
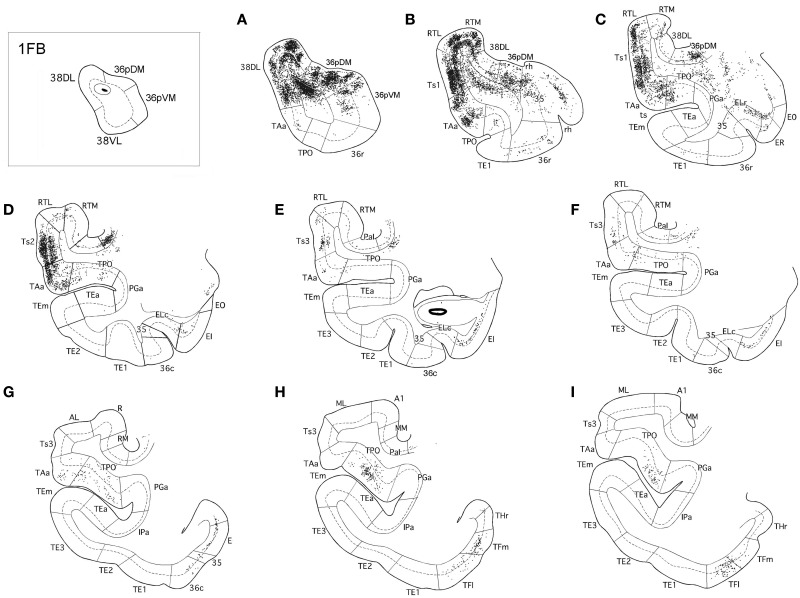
**Rostrocaludal (A–I) series of coronal sections through the temporal lobe illustrate the density and laminar distribution of label in temporal cortex after retrograde tracer injections in area 38_DL_ of the dorsolateral temporal pole in a representative case (1FB)**. The retrograde tracer injection is indicated in the inset.

**Figure 6:**
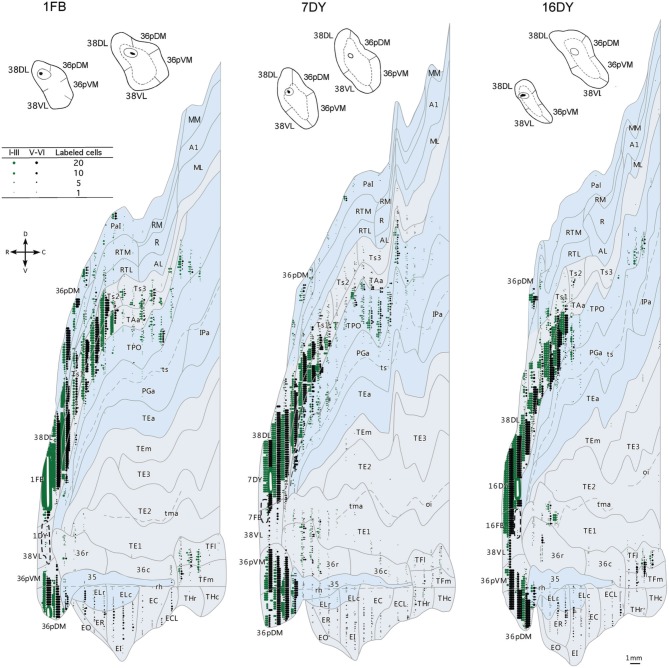
**Two-dimensional unfolded maps of the temporal cortex show the topographical distribution of retrogradely labeled cells after injections in area 38_DL_**. Retrograde label in layers I–III (in green) and in layers V–VI (in black) indicate the laminar distribution of label (see inset for symbols). Injection sites are indicated in coronal sections and in the two-dimensional maps (solid white lines). Adjacent injection sites (dotted lines) are also represented in the maps. Note the higher density of label in areas Ts1, TAa, and TPO compared with the subdivisions of area TE.

The three retrograde tracer injections placed in area 38_DL_ resulted in extensive retrograde labeling of neurons in adjacent areas of the rostral STG. As Figures 5, 6 show, layers II–III and V–VI of areas Ts1 and rostral TAa contained the largest density of labeled neurons accounting for up to 70% of the temporal cortical input to this area. The density of retrograde label decreased substantially at more caudal levels in area Ts3 (Figures 5, 6, Table 1). Areas RTL and RTM contained up to 11% of the temporal cortex input to 38_DL_ and distributed across layers II–III and V–VI rostrally, and in layers II–III more caudally. As illustrated in Figures 5, 6, the density of retrograde label was high in the multimodal area of the dorsal bank of the ts; specifically at rostral and mid-levels of area TPO (layers II–III and V–VI), accounting up to 21% of the total temporal cortex input to 38_DL_. The density of retrograde label in area TPO decreased medially toward the fundus of the ts (area PGa) and caudally at the level of Ts2-Ts3. Visual processing area TE1 of the inferior temporal cortex had very modest retrograde label (2% of temporal cortex input) located in layers II–III and V–VI (Table 1, Figure 6).

**Table 1:** **Percentage of labeled neurons in the architectonic areas of the rostral STG (RTM, RTL, Ts1-3, TAa, TPO, PGa, IPa), inferior temporal gyrus (TE), EC, areas 35 and 36 of PRC, and areas TH and TF of PHC**.

**Injection site**	**Case**	**RTM/RTL**	**Ts1**	**Ts2**	**Ts3**	**TAa**	**TPO**	**PGa**	**IPa**	**TE1-a**	**EC**	**PRC**	**PPH**
38_DL_	1FB	7^a^ (11)^b^	30 (38)	6 (7)	1 (2)	20 (26)	6 (8)	0 (0.3)	0	0	3 (3)	2 (2)	2 (3)
	7DY	2 (3)	18 (29)	2 (3)	0.2 (0.2)	17 (27)	13 (21)	2 (3)	0	1 (2)	2 (3)	6 (9)	1 (1)
	16DY	0.1 (0.2)	26 (44)	0.3 (0.5)	0	16 (26)	10 (16)	1 (1)	0	1 (2)	2 (3)	2 (3)	2 (4)
38_VL_	1DY	0.3 (0.3)	6 (8)	0	1 (1)	14 (17)	28 (33)	6 (7)	0	14 (16)	2 (2)	11 (13)	3 (3)
	6FB	0.1 (0.2)	3 (5)	6 (9)	0.1 (0.1)	6 (9)	14 (20)	10 (14)	0	10 (13)	3 (4)	12 (17)	9 (12)
	14DY	3 (4)	3 (3)	2 (2)	0.4 (0.4)	12 (14)	19 (23)	9 (11)	0	11 (13)	0.2 (0.2)	8 (10)	0.2 (0.2)
	8DY	0.1 (0.1)	0.4 (0.6)	0.3 (0.4)	0	17 (22)	33 (43)	13 (17)	0	9 (12)	0 (0.8)	3.8 (5)	0.6 (0.5)
	2FB	0 (0.4)	4 (6)	0 (0.3)	0	6 (9)	18 (27)	10 (15)	0	5 (8)	3 (4)	20 (30)	0 (1)
38_DL_/38_VL_ border	7FB	2 (2)	5 (6)	1 (1)	1 (1)	10 (11)	19 (22)	9 (11)	0	14 (16)	0.5 (0.6)	24 (29)	0.2 (0.2)
	3FB	2 (2)	7 (7)	4 (5)	1 (1)	15 (16)	31 (34)	6 (6)	0.1 (0.1)	9 (10)	1 (2)	12 (14)	5 (5)
	16FB	0.3 (0.4)	2 (2)	3 (4)	1 (1)	14 (17)	19 (22)	19 (22)	0.3 (0.3)	12 (15)	2 (2)	12 (14)	2 (3)

a *Percent of retrogradely labeled neurons of the total labeled neurons in the whole cerebral cortex*.

b *Percent of retrogradely labeled neurons of the total labeled neurons in the temporal cortex*.

**Figure 7:**
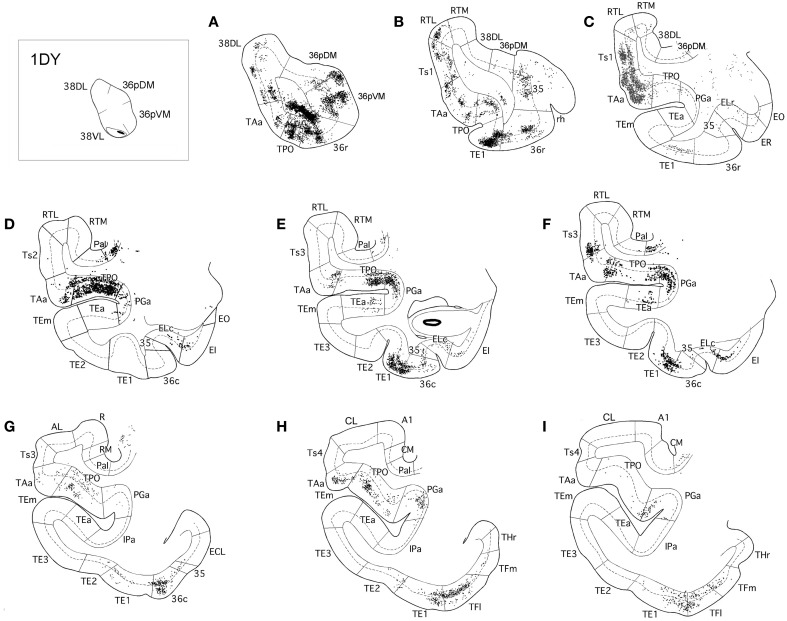
**Rostrocaudal (A–I) coronal sections through the temporal lobe illustrate the laminar and density pattern of the projection from temporal cortex to area 38_VL_**. The retrograde tracer injection site is indicated in the inset.

**Figure 8:**
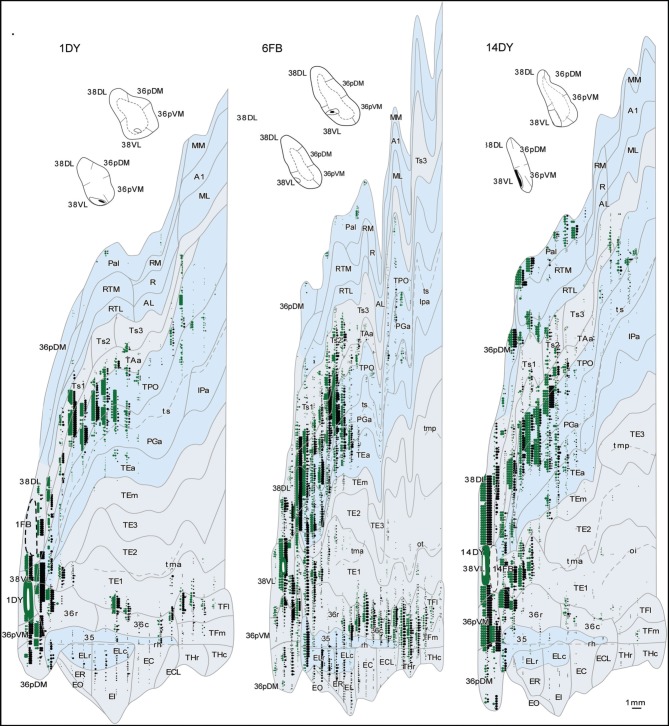
**Two-dimensional maps of the temporal cortex show the topographical distribution of retrograde label after retrograde tracer injections in area 38_VL_**. Note the low density of retrograde label in areas Ts1 and Ts2 and, by comparison, the higher density of label in areas TPO, PGa, TE, and in 36 of PRC, compared with more dorsolateral injections in area 38_DL_. Symbols and abbreviations as in previous figures.

In contrast, as Figures 7, 8 show, injections in area 38_VL_ (*n* = 5), yielded the largest amount of labeled cells in multimodal area of the dorsal bank of the ts, accounting for up to 60% of the temporal input to this area. Within the cortex of the dorsal bank of the ts, layers II–III and V–VI of area TPO had the highest density of retrograde label (43% of the temporal input), followed by area PGa in the fundus of the ts (17%, Table 1, Figure 7). This projecting region of the ts continued laterally and encroached area TAa (22%) in the gyral surface of the rostral STG (Figure 7). The density of retrograde label decreased in areas Ts1, RTL, and RTM (Figures 7, 8). However, visual processing area TE had high density of retrograde label (up to 16% of the temporal input). Label in area TE was concentrated primarily in the rostral portion of subareas TEa and TEm in the ventral bank of the ts, and in two large patches in area TE1 on the gyral surface, one rostral and another one located more caudally.

**Figure 9:**
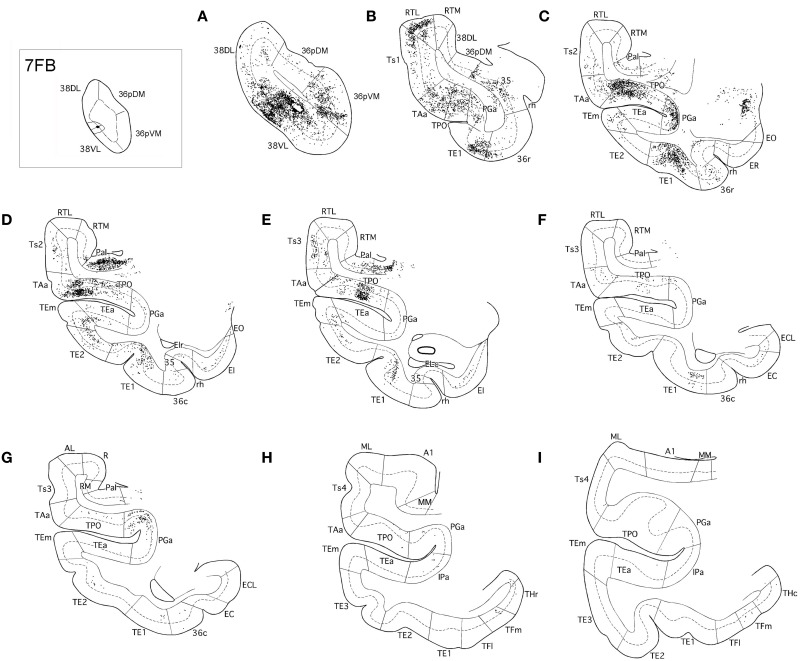
**Rostrocaudal (A–I) coronal sections through the temporal lobe illustrate the density and the laminar distribution of retrograde label in temporal cortex after injections at the 38_DL/VL_ boundary**. The retrograde tracer injection site is indicated in the inset.

**Figure 10:**
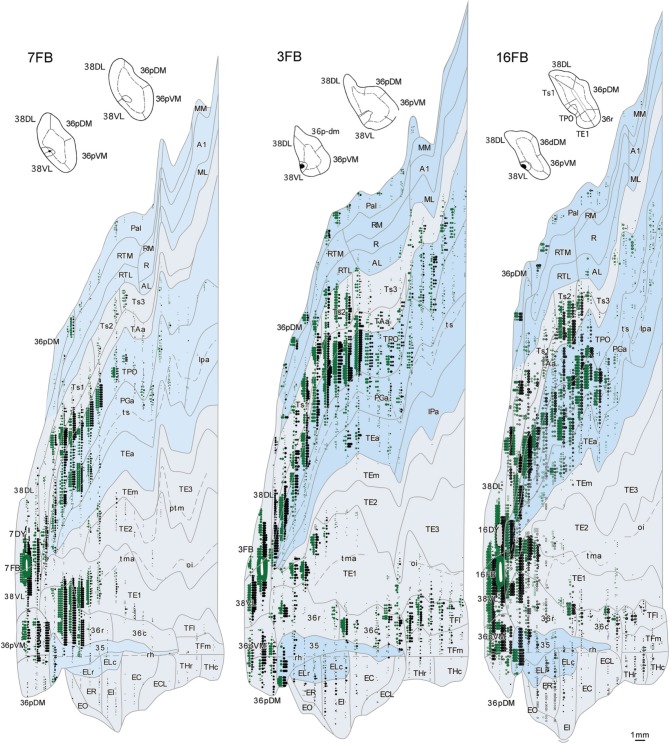
**Two-dimensional maps of the temporal cortex show the topographical distribution of retrograde label in temporal cortex after injections at the 38_DL/VL_ boundary**. Note that in addition to the high density of retrograde label in area Ts1 and Ts2, there is a higher density of retrograde label in areas TAa, TPO and PGa, TE and area 36 of PRC compared with more dorsolateral injections in area 38_DL_.

The retrograde injections in area 38 near the 38_DL_/38_VL_ boundary (*n* = 3) labeled neurons that took a transitional pattern of distribution between that seen after the more dorsal and ventral injections in 38. In one hand, as Figures 9, 10 show, injections near the 38_DL_/38_VL_ boundary resulted highest density of retrograde label in layers II–III and V–VI of the multimodal areas TPO and PGa of the dorsal bank of the ts, accounting for up to 40% of the temporal cortex input to this area, with the heaviest contribution from area TPO (34% of the temporal input) followed by area PGa (up to 17%). On the other hand, the next heaviest projection originated similarly in terms of densities from both auditory and visual processing areas; such as the rostral part of area TAa (17%) and areas TE1-2, TEa, and TEm (16%). Areas Ts1 (7%), Ts2 (5%), Ts3 (1%), and RTL/RTM (2%) of the STG also contained retrograde label. Like in previous cases, the density of retrograde label decreased progressively at more caudal levels in all areas (Figures 9, 10).

#### Anterograde injections in areas Ts3, Ts2, and RTL

**Figure 11:**
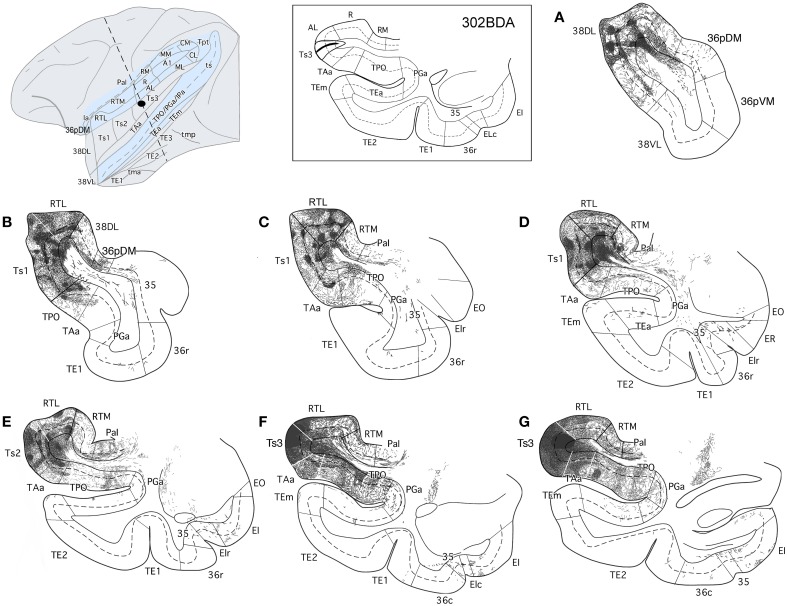
**The projection from area Ts3 of STG to the temporal cortex is shown in rostrocaudal (A–G) coronal sections through the temporal lobe after a BDA injection in area Ts3**. Labeled fibers course rostrally to areas Ts2, Ts1, and the temporal pole areas 38_DL_ and 36_pDM_, and laterally to areas TAa and TPO. Note the scarce projection from area Ts3 to the medial temporal cortex.

**Figure 12:**
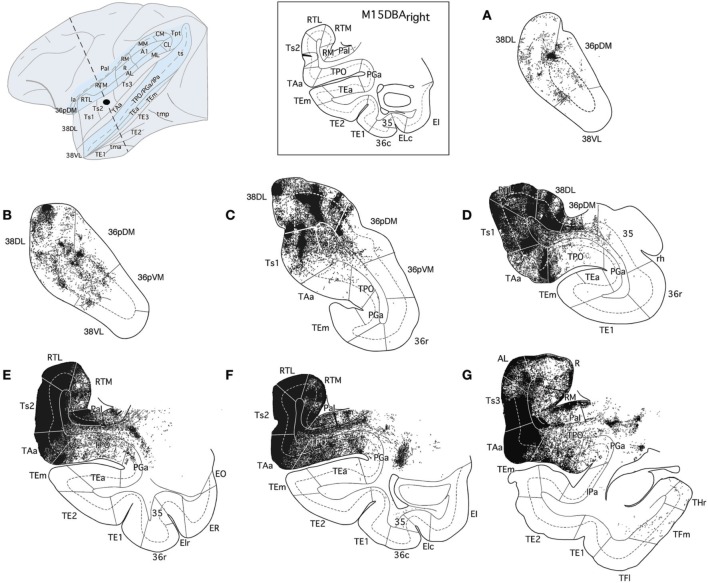
**The projection from area Ts2 of STG to the temporal cortex is illustrated in rostrocaudal (A–G) coronal sections through the temporal lobe after a BDA injection in area Ts2**. Anterogradely labeled fibers coursed rostrally toward area Ts1 and areas 38_DL_ and 36p_DM_ of the temporal pole and laterally to areas TAa and TPO in the cortex of the dorsal bank of the ts. Note that the direct projection from area Ts2 to the medial temporal cortex is very scarce.

The anterograde injections in areas Ts2 and Ts3 (Figures 11, 12, respectively) yielded similar patterns of anterograde label in the temporal lobe. Both injections resulted in dense bundles of labeled axons with termination in layers II–III and V–VI of the neighboring areas Ts1, RTL, RTM, and the parainsular area PaI as well as in areas TAa and TPO within the cortex of the dorsal bank of the ts. These bundles of labeled axons coursed rostrally within the temporal lobe white matter with extensive termination label in layers II–III and V–VI of area 38_DL_ and area 36_pDM_ (Figures 11, 12). Anterograde labeled fibers appeared to form columns in the temporopolar cortex, but only occasionally in areas Ts1, RTL, RTM, PaI, TPO, and none were observed in area TAa (Figures 11, 12).

**Figure 13:**
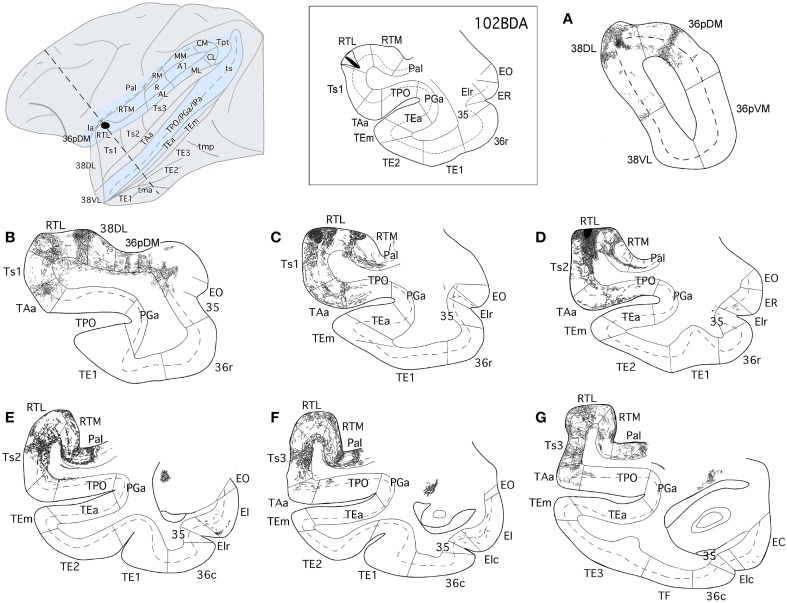
**The projection from area RTL to the temporal cortex is shown in rostrocauldal (A–G) coronal sections through the temporal lobe after a BDA injection in area RTL**. The anterograde projection courses rostrally within the STG white matter to end in the areas Ts2, Ts1, TAa, and TPO and reaches as far rostal as areas 38_DL_ and 36p_DM_ of the temporal pole. Note that the direct projection from area RTL to the medial temporal cortex is very scarce.

The BDA injection in area RTL of the rostral STP (Figure 13) labeled axons that coursed medially toward the adjacent area RTM and the parainsular cortex (PaI), where terminal label took a columnar-like appearance across layers II–III and V–VI. Another bundle of labeled fibers coursed laterally to areas Ts1-Ts3, TAa, and, to less so to area TPO. Labeled fibers continued rostrally toward the temporal pole to terminate primarily in layers II–III and V–VI of areas 38_DL_ and 36_pDM_ in the dorsal temporopolar cortex (Figure 13A).

It is worth noting that none of these anterograde injections in rostral STG areas resulted in any substantial anterograde label in the medial temporal cortex, whereas anterograde label was found in the temporal pole and multimodal areas of the ts. However, the medial temporal cortical areas that receive this scarce projection also receive projections from 38_DL_ (i.e., E_Lr_, E_R_, and E_I_ and areas TH and TF, see next section).

### Projections from the dorsolateral temporal pole (38_DL_) to medial temporal cortex

#### Temporal pole intrinsic connections

**Figure 14:**
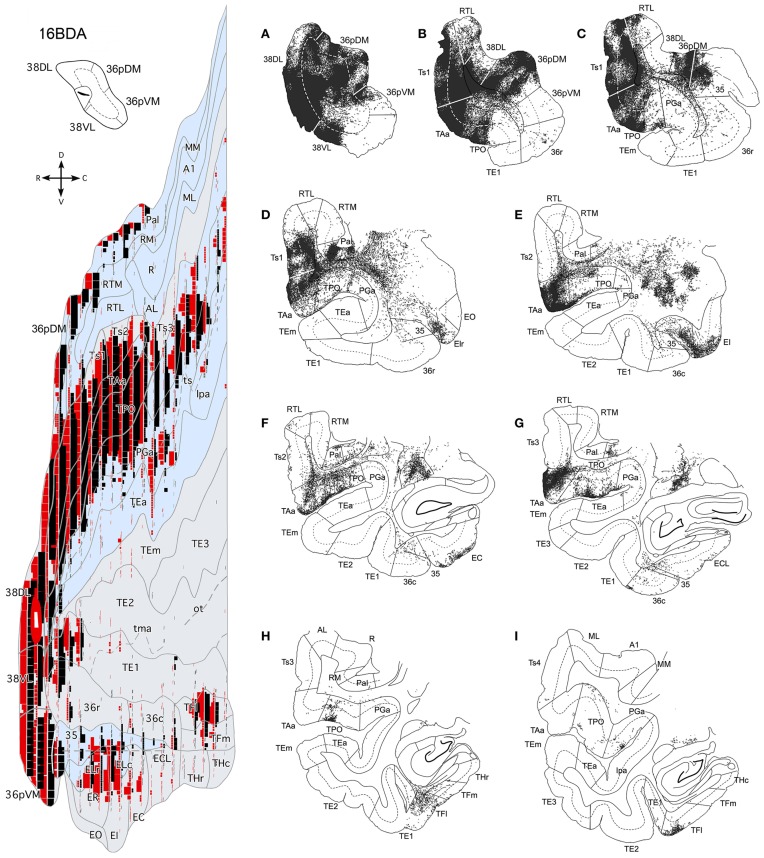

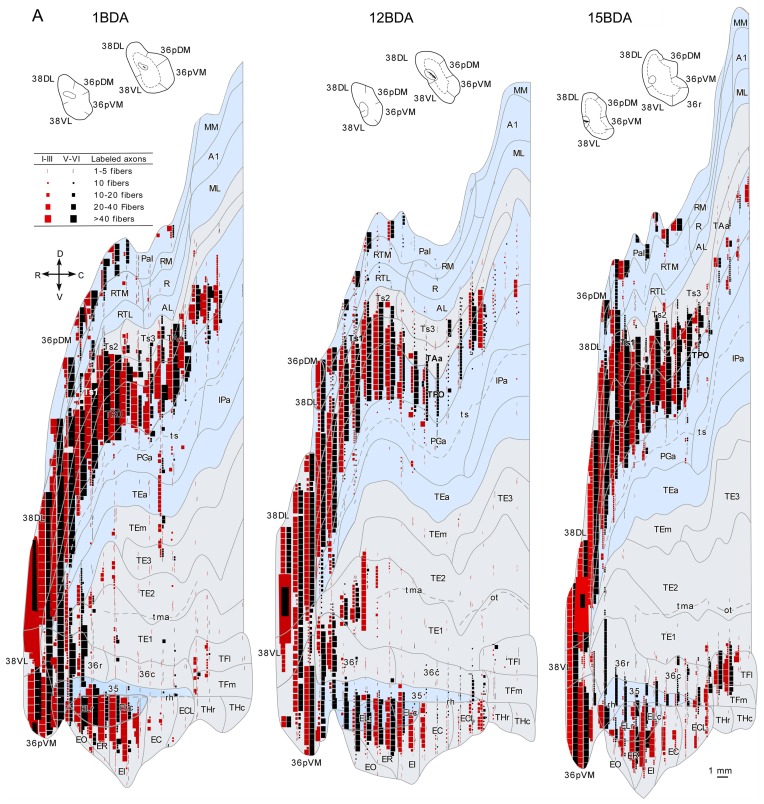
**Anterograde projection from area 38_DL_ to medial temporal cortex. (A)** Two-dimensional map and rostrocaudal coronal sections through the temporal lobe of one of the cases illustrate the topographic, density, and laminar distribution of label in the temporal cortex. Note the high density of label in the outer layers of the entorhinal cortex. **(B)** Two-dimensional maps illustrate the distribution of anterograde label in the temporal cortex after BDA injections in area 38_DL_. Anterogradely labeled axons were primarily found in areas 36_pDM_, EC, 35 of PRC, and PHC areas TH and TF. In contrast, area 36 of PRC contained almost no labeled fibers.

Anterograde tracer injections in area 38_DL_ yielded a high density of labeled axons and terminals in layers in layers II–III and V–VI of the adjacent area 36_pDM_ and extending more moderately 36_pVM_, suggesting a pattern of high density of local connectivity within the most dorsal subdivisions of the temporal pole and less so with the more ventral subdivisions (Figure 14).

#### Entorhinal cortex (EC)

While density of anterograde label in the EC olfactory division (E_O_) was scarce, it increased substantially in layers I–III and V–VI of the rostral-lateral EC (E_R_, E_Lr_, and E_Lc_) and then decreased again caudally and medially in the subdivisions E_I_, E_C_, and E_CL_ with label primarily in layers I–III (Figure 14). Anterograde label tended to occupy all layers of the EC when label was densest and layers II–III when label was moderate to light.

It is interesting to note that the laminar and topographical distribution of label in EC was different after the retrograde and anterograde injections in 38_DL_. In contrast to the rostral-lateral distribution of anterograde label in EC, retrograde label was almost absent in the lateral divisions (E_Lr_ and E_Lc_) and distributed medially in E_R_, E_I_, E_C_, and E_CL_ (compare Figures 5, 6 with Figure 14). In terms of laminar distribution, retrograde label, was more restricted and concentrated primarily in layers V–VI of the EC projecting subdivisions (E_R_, E_I_, E_C_, and E_CL_).

#### Perirhinal cortex (PRC)

As shown in Figure 14, area 36_r_ had a modest density of anterograde label and was located in its most rostral portion and distributed across layers. Area 36_c_ had only very light density of labeled fibers that often continued with label in area TF_l_ of the posterior parahipocampal cortex. In contrast, area 35 of PRC, along the fundus of the rhinal sulcus, had moderate density of labeled fibers primarily in layers V and VI. It is worth noting that the topographical distribution of anterograde and retrograde label in areas 35 and 36 of PRC after 38_DL_ injections was similar.

#### Posterior parahippocampal cortex (PHC)

Anterograde label was found in the rostral half of areas TH and TF, primarily in layers I–III and V–VI of the lateral division of area TF (TF_l_). Anterograde label became progressively lighter and more restricted to layers I–III more caudally in TF_m_ and area TH (Figure 14). It is worth noting that the topographical distribution of anterograde and retrograde label in areas TH and TF of PHC after 38_DL_ injections was similar.

## Discussion

**Figure 15:**
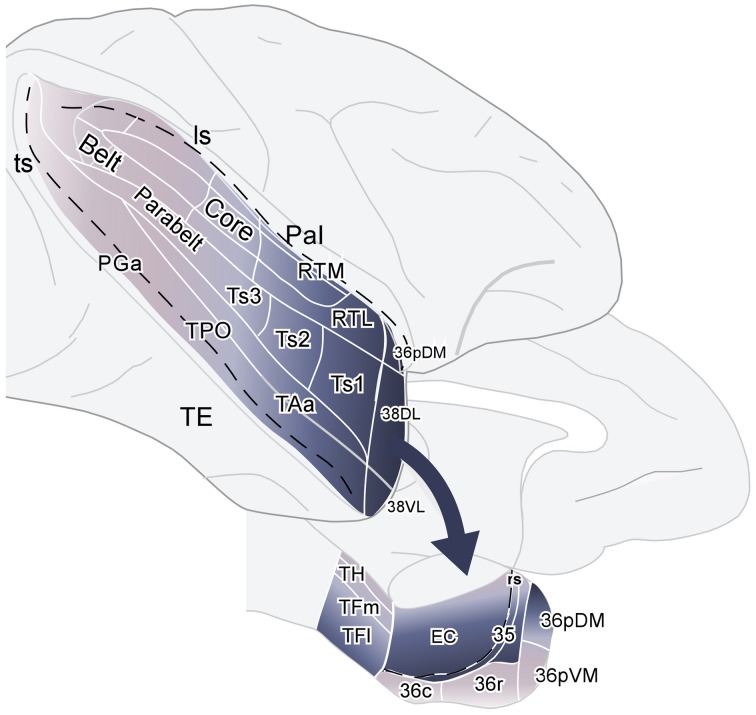
**Schematic diagram summarizing the projections from auditory association areas of the rostral superior gyrus to the dorsolateral temporal pole area 38_DL_ and from this area 38_DL_ to the subdivisions of the medial temporal cortex**. The main entry of auditory information to the medial temporal cortex courses via EC, area 35 of PRC, and areas TH and TF of PHC, but bypasses most of area 36 of PRC.

The aim of this study was to determine if or how highly processed auditory information might enter the medial temporal cortex. Our results showed first, that about 70% of the total temporal input to area 38_DL_ of the dorsolateral temporal pole originated in the auditory processing areas of Ts1 and TAa of the rostral STG and area RTL of the rostral STP. Second, area 38_DL_ sends this information to EC, area 35 of PRC, and areas TH-TF of the PHC. Third, the projection to area 36 of PRC are restricted to the most rostral part of its rostral subdivision 36_r_ and the most caudal portion of 36_c_; this caudal patch of cells was often continuous with that of TF_l_ (see summary in Figure 15). Fourth, area 38_DL_ of the temporal pole receives a proportion of its input from polysensory areas of the cerebral cortex (i.e., dorsal bank of the ts, orbital frontal, medial frontal, agranular insular, and medial temporal cortices), and therefore, this area may integrate auditory information with inputs from other sensory modalities. We discuss our results with previous studies of auditory processing within the STG and STP and conclude with the implications of our own results on the anatomical organization of memory pathways for audition.

### Sensory domains in the temporal pole

The importance of the projections from the rostral part of the STG to the temporal pole for the processing of higher order auditory information was first suggested by Jones and Powell (1970) and supported by Moran et al. (1987). They showed that, whereas the medial subdivisions of the temporal pole receive primarily olfactory and limbic input, the dorsolateral temporal pole (38_DL_ here) receives input from auditory processing areas. Later anatomical studies suggested an anatomical schema whereby anterior subdivisions of the auditory belt send projections to progressively more anterior portions of the STG (Seltzer and Pandya, 1978; Galaburda and Pandya, 1983; Cipolloni and Pandya, 1989; Kaas and Hackett, 2000). This stream of connections would course rostrally to reach the temporal pole (Markowitsch et al., 1985), in particular, the dorsolateral aspect of the temporal pole (Moran et al., 1987, our own results). Our results, therefore, support previous studies and add that auditory input represents about 50% of the total cortical input and 70% of the total temporal cortex input to area 38_DL_ of the dorsolateral temporal pole.

### The ventral auditory stream

Whether there is a unique auditory ventral stream within the STG directed rostrally or an additional one directed medio-laterally toward the gyral convexity and the cortex of the STG remains still an open question (Bendor and Wang, 2008, see discussion in Kikuchi et al., 2010; Tanji et al., 2010). Although our study addressed primarily the rostral end of the ventral stream, our results reinforce the hypothesis that downstream projections within the rostral STG might be organized in two main parallel streams. As illustrated in Figures 11–13, anterograde injections in areas Ts3, Ts2, and RTL labeled axons that course toward area 38_DL_ of the dorsolateral temporal pole in a rostrally directed stream, but these injections also labeled axons that course laterally to areas TAa and TPO of the gyral convexity and dorsal bank of the ts. Despite the unknown mechanisms underlying the stimulus processing by both streams, fMRI and electrophysiological data suggest that the adjacent areas Ts1 and Ts2 are especially important for encoding complex sounds, including conspecific calls in monkeys (Petkov et al., 2008; Kikuchi et al., 2010; Fukushima et al., 2014). Although fMRI data call-activation areas are located in areas Ts1-2, PET studies in primates have shown that the dorsal aspect of the temporal pole (area 38_DL_ in this study) is especially responsive to species-specific calls (Poremba et al., 2003, 2004; Gil-da-Costa et al., 2006). The differences in functional activation in fMRI vs. PET reports might be explained by differences in vulnerability to scanning artifacts. A comparative PET-fMRI study in humans showed speech-activated regions in the temporal pole region using PET but not fMRI, suggesting that whereas fMRI signal in the temporal pole is more vulnerable to artifacts, PET can detect activity in this region more reliably (Devlin et al., 2000). The authors also suggest that fMRI requires to adapt data acquisition paradigms and/or the use of ROI analysis to match PET sensitivity. This leaves the doors open to compare between primate PET and fMRI studies on complex auditory stimulus processing.

However, the cortical network for recognition of species-specific monkeys calls might be a large one of which the dorsolateral temporal pole (area 38_DL_) is only one part. According to functional 2-deoxyglucose data, auditory processing includes the entire STG, and some regions of the frontal, parietal, and medial temporal cortical areas (Poremba et al., 2003). In line with this, fMRI data suggests that area 38_DL_ of the dorsolateral temporal pole (Gil-da-Costa et al., 2004; Poremba et al., 2004; Petkov et al., 2008), inferior frontal and parietal regions (possible analogs of Broca's and Wernicke's areas, Gil-da-Costa et al., 2006), area 32 of the medial frontal cortex, amygdala, and hippocampus are especially important for the processing of species-specific calls (Gil-da-Costa et al., 2004). All the components of the network have connections with area 38_DL_ (Muñoz et al., 2003, present results) from which information is forwarded to 36_pDM_, EC, area 35 of PRC and posterior areas TH and TF of PHC and to the most rostral portion of area 36_r_. This rostral STG-38_DL_-EC/PRC/PHC pathway, although not functionally enough to support long-term recognition of purely auditory information as tested with DMS tasks, it may still be important for the storage of complex auditory information in rhesus monkeys, especially con-specific calls (Wich and de Vries, 2006; Ng et al., 2009).

A recent study reported neurons in the dorsolateral temporal pole (area 38_DL_ here) that responded to task-relevant events in a delayed matching task, with some neuronal responses associated with accuracy in recognition performance in a DMS task (area dTP in Ng et al., 2014). Some neurons in area 38_DL_ showed match suppression responses similar to those observed in the visual object identification pathway located in the ventral part of the temporal pole (area 38_VL_ here, Desimone, 1996; Nakamura and Kubota, 1996). This suggests that the dorsolateral temporal pole might be an important area for memory encoding.

It is important to mention here the case of tactile memory. Even though monkeys and humans retain tactile information in mind efficiently for long delays (Goulet and Murray, 2001; Bigelow and Poremba, 2014), the projection from higher order somatosensory areas that process touch in the granular insula is restricted to area 35 of PRC (Murray and Mishkin, 1984; Schneider et al., 1993; Friedman et al., 1986). However, the anatomical pathway for touch despite of being restricted, just like the auditory one, it appears to be sufficient to hold tactile information in mind long enough as to be transferred in to long-term memory in primates, but also in humans (Bigelow and Poremba, 2014). However, a possible explanation for this is that tactile information is *translated* internally to vision and gets remembered by means of using the visual memory pathway. This is a working hypothesis that calls for further research.

### Auditory memory pathway

The rostral part of STG (38_DL_, Ts1, TAa) and area TPO in the dorsal bank of the ts sends information directly to EC (Amaral et al., 1983, see review in Mohedano-Moriano et al., 2007; Insausti and Amaral, 2008). However, with the exception of a dense projection from area TPO, these areas of the rostral STG only send a meager projection to areas 35 and 36*_r/c_* of PRC (Suzuki and Amaral, 1994b; Kondo et al., 2003; Muñoz et al., 2003). There is another minor entry of auditory input to the medial temporal cortex via a small projection from the caudal part of STG to area TH of PHC (Tranel et al., 1988; Suzuki and Amaral, 1994b). Our results show that the areas that form the rostral STG project mainly to area 38_DL_, which in turn projects to EC, area 35 of PRC, and areas TH and TF of PHC. However, and in striking disparity with the pathway important for visual memory (TE-PRC-EC), this projection bypasses most of area 36*_r/c_* of PRC. This finding in particular might offer an explanation, at least in part, of the poor recognition memory ability of rhesus monkeys in audition. An explanation that might be extensive to the poorer ability for auditory memory in humans compared with touch and vision (Bigelow and Poremba, 2014).

## Conclusion

We have shown that area 38_DL_ receives 70% of its cortical input from the auditory association region of the rostral STG, with a substantial input from the polysensory areas of the ts, medial frontal, orbitofrontal, insular, and medial temporal cortices. These results are consistent with lesion and functional imaging in rhesus monkeys suggesting that, among other functions, the dorsolateral temporal pole processes complex auditory stimuli (including species-specific calls). Area 38_DL_ sends heavy projections to the EC, area 35 of PRC and areas TH and TF of PHC, but bypasses most of area 36*_r/c_* of PRC. This anatomical arrangement may contribute to our understanding of the poor auditory memory of rhesus monkeys.

### Conflict of interest statement

The authors declare that the research was conducted in the absence of any commercial or financial relationships that could be construed as a potential conflict of interest.

## Abbreviations

35: area 35 of the perirhinal cortex (Brodmann, 1909)
36_r_: rostral division of area 36 of the perirhinal cortex (Insausti et al., 1987)
36_c_: caudal division of area 36 of the perirhinal cortex (Insausti et al., 1987)
36_pDM_: dorsal medial division of the temporal pole
36*_pVM_*: ventral medial division of the temporal pole
38*_DL_*: dorsal lateral division of the temporal pole
38_VL_: ventral lateral division of the temporal pole
A1: area A1 (Kaas and Hackett, 2000)
AL: area AL (Kaas and Hackett, 2000)
amts: anterior middle temporal sulcus
cc: corpus callosum
CL: area CL (Kaas and Hackett, 2000)
CM: area CM (Kaas and Hackett, 2000)
DM: area DM (Kaas and Hackett, 2000)
EC: entorhinal cortex
E_C_: caudal subfield of EC (Amaral et al., 1987)
E_CL_: caudal limiting subfield of EC (Amaral et al., 1987)
E_I_: intermediate subfield of EC (Amaral et al., 1987)
E_Lc_: lateral caudal subfield of EC (Amaral et al., 1987)
E_Lr_: lateral rostral subfield of EC (Amaral et al., 1987)
E_O_: olfactory subfield of EC (Amaral et al., 1987)
E_R_: rostral subfield of EC (Amaral et al., 1987)
Ia: insula
IPa: area IPa (Seltzer and Pandya 1978, 1989)
la: lateral sulcus
MM: area MM (Kaas and Hackett, 2000)
PaI: Parainsular cortex
PHC: areas TH and TF of the parahippocampal cortex
PGa: area PGa (Seltzer and Pandya 1978, 1989)
pmts: posterior middle temporal sulcus
PRC: areas 35 and 36 of the perirhinal cortex
R: area R (Kaas and Hackett, 2000)
RM: area RM (Kaas and Hackett, 2000)
rs: rhinal sulcus
RT: area RT (Kaas and Hackett, 2000)
RTL: area RTL (Kaas and Hackett, 2000)
RTM: area RTM (Kaas and Hackett, 2000)
STG: superior temporal gyrus
STP: superior temporal plane
ts: superior temporal sulcus
TAa: area Tpt (Seltzer and Pandya 1978, 1989)
TE: area TE (Von Bonin and Bailey 1947)
TF: area TF (Von Bonin and Bailey 1947)
TF_l_: lateral division of area TF (Insausti et al., 1987)
TF_m_: lateral division of area TF (Insausti et al., 1987)
TH: area TH (Von Bonin and Bailey 1947)
TH_c_: caudal division of area TH (Insausti et al., 1987)
TH_r_: rostral division of area TH (Insausti et al., 1987)
TPC: temporal pole cortex
TPO: area TPO (Seltzer and Pandya 1978, 1989)
Tpt: area Tpt (Seltzer and Pandya 1978, 1989)
Ts1: area Ts1 (Seltzer and Pandya 1978, 1989)
Ts2: area Ts2 (Seltzer and Pandya 1978, 1989)
Ts3: area Ts3 (Seltzer and Pandya 1978, 1989).
